# Tubular aggregates caused by serine active site containing 1 (*SERAC1*) mutations in a patient with a mitochondrial encephalopathy

**DOI:** 10.1111/nan.12190

**Published:** 2015-03-04

**Authors:** Yehani Wedatilake, Vincent Plagnol, Glenn Anderson, Simon M. L. Paine, Peter T. Clayton, Thomas S. Jacques, Shamima Rahman

**Affiliations:** ^1^ Genetics and Genomic MedicineUCL Institute of Child HealthLondonUK; ^2^ Developmental Biology and CancerUCL Institute of Child HealthLondonUK; ^3^ UCL Genetics InstituteLondonUK; ^4^ Department of HistopathologyGreat Ormond Street Hospital NHS Foundation TrustLondonUK; ^5^ Metabolic DepartmentGreat Ormond Street Hospital NHS Foundation TrustLondonUK

Tubular aggregates (TAs) are cytoplasmic aggregates of membranous tubules derived from the sarcoplasmic reticulum and usually 50–70 nm in diameter [1]. They may be seen in a range of genetic myopathies, including gyrate atrophy caused by ornithine aminotransferase deficiency, periodic paralysis and two disorders of glycosylation caused by mutations in the *GFPT1* and *DPAGT1* genes [2, 3]. In addition, they may occur as a minor or inconsistent feature in a wide range of myopathies or be the predominant feature in idiopathic or congenital myopathies [4]. However, the mechanisms underlying TA formation remain poorly understood. We now report the development of TAs in a patient with a lipid remodelling disorder, providing new insight into the formation of TAs.

We report the case of a 13‐year‐old boy with a progressive mitochondrial encephalopathy in the Leigh syndrome spectrum. He is the first child of healthy unrelated Bengali parents and has an unaffected younger sister. He initially presented on day one of life with transient neonatal lactic acidosis and hyperammonaemia, which resolved with conservative management. Muscle biopsy in the neonatal period revealed undetectable activity of succinate‐cytochrome *c* reductase [respiratory chain (RC) complexes II + III], with normal activities of complexes I and IV (Table 1). By 6 months, poor feeding and growth were apparent, and at 18 months, he was treated with hearing aids for bilateral sensorineural hearing loss (SNHL). At 2 years, he developed challenging behaviour, associated with developmental regression. From 3 years, he had recurrent chest infections, progressing to bronchiectasis. At 6.5 years, he had an episode of encephalopathy with status dystonicus requiring invasive ventilation. At 9 years, Nissen fundoplication was performed to treat severe gastro‐oesophageal reflux, and a Percutaneous Endoscopic Gastro‐Jejunostomy (PEG‐J) was inserted. Muscle biopsy was repeated at 12 years at the time of a gastrostomy revision and revealed that RC activities were now essentially normal, other than borderline reduction of cytochrome *c* oxidase (COX, complex IV) activity (Table 1). RC activities were normal in cultured skin fibroblasts (Table 1). Now 13 years, he has microcephaly, optic atrophy, severe dystonia and self‐harm involving biting and scratching. He receives prophylactic antibiotics to prevent further respiratory infections and requires regular suction and intermittent face mask oxygen. The results of metabolic investigations are summarised in Table 1. Neuro‐imaging demonstrated abnormal signal in the striatum bilaterally and associated atrophy in the caudate heads.

**Table 1:** Metabolic investigations in a patient with SERAC1 deficiency

Investigation	Results
Blood lactate	Ranged from 5.7–7.4 mmol/L (reference range 0.7–2.1)
CSF lactate	2.0 mmol/L (normal <2)
Urine organic acids	Grossly raised lactate, pyruvate and 2‐hydroxybutyrate with moderately raised 2‐hydroxyisovalerate and mildly raised 2‐oxo‐isocaproate.
	Strongly raised 3‐methylglutaconate with moderately raised 3‐methylglutarate
Plasma amino acids	Increased levels of
	Alanine 587 μmol/L (150–450)
	Glutamine 1143 μmol/L (480–800)
	Tyrosine 394 μmol/L (30–120)
	Proline 569 μmol/L (85–290)
Fibroblast enzyme activities	Pyruvate dehydrogenase activity 0.85 nmoles/mg
	Protein/min (reference range of 0.7–1.1)
	Complex II + III 0.146 (0.07–0.243)
	Complex IV 0.016 (0.007–0.036)
Muscle respiratory chain enzyme activities	At age 15 days
	Complex I 0.101 (0.104–0.268)
	Complex II and III undetectable (0.040–0.204)
	Complex IV 0.015 (0.014–0.034)
	At age 12 years
	Complex I 0.149 (0.104–0.268)
	Complex II + III 0.103 (0.040–0.204)
	Complex IV 0.013 (0.014–0.034)

CSF, Cerebrospinal fluid.

**Figure 1:**
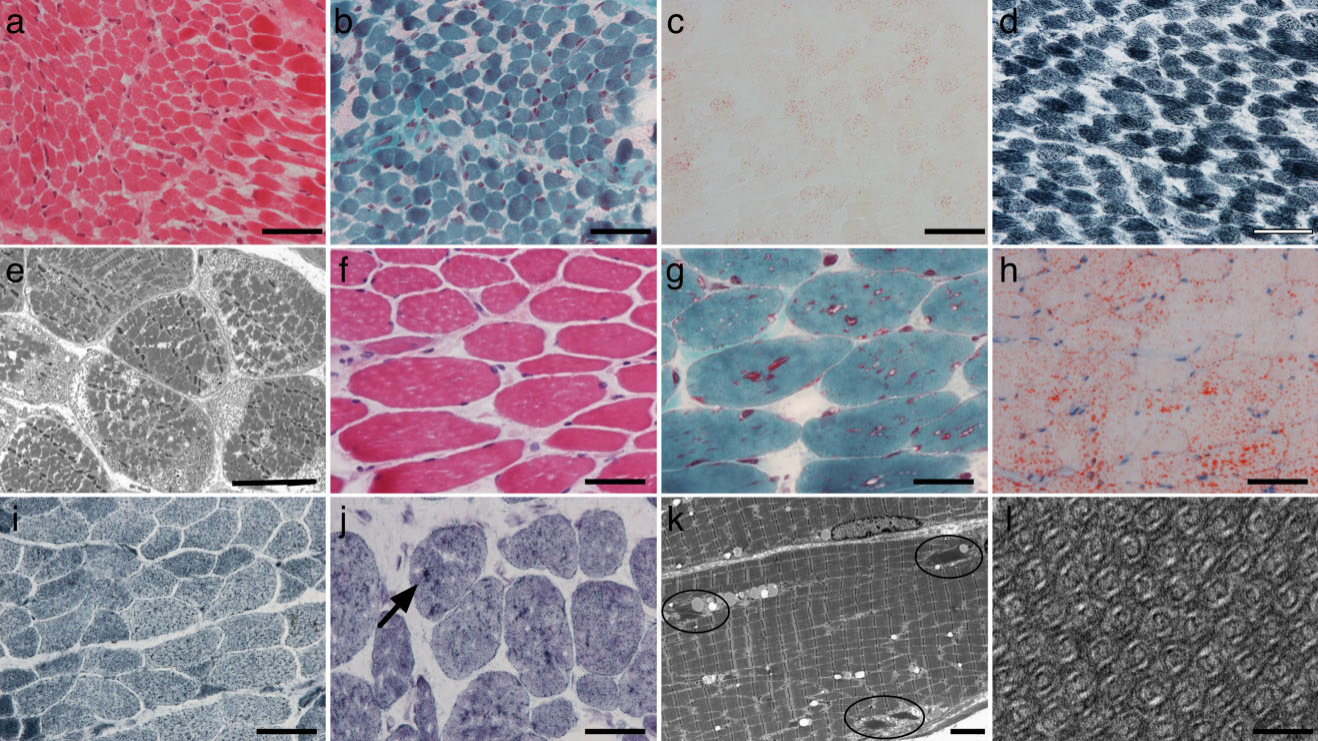
Muscle biopsies taken at 15 days (**a**–**e**) and 12 years of age (**f**–**i**). At 15 days, the biopsy did not show significant diagnostic abnormalities (**a**, H&E; **b**, Gömöri trichrome; **c**, Oil red O; **d**, NADH‐TR). Ultrastructural examination revealed prominent nonmembrane‐bound glycogen in the sarcoplasm (**e**). Specifically, there were no tubular aggregates. In contrast, at 12 years of age, there were small, predominantly angular, fibres and scattered fine vacuolation and occasional deposits highlighted by the Gömöri stain (**f,** H&E; **g**, Gömöri trichrome). There was excess lipid for the age (**h**, Oil red O). There was a little granularity on NADH‐TR staining (**i**) but without well‐defined aggregates. Histochemical staining for adenylate deaminase (**j**) showed scattered dense staining structures (arrow). Electron microscopy revealed very frequent collections of tubular aggregates, many of which are relatively small (indicated by the circles) (**k**). These showed a typical pattern of concentric double‐lumen tubules (**l**). Scale bars, **a**, **b**, **c, d**, **f**, **g**, **h**, **i**, **j**: 50 μm; **e**: 10 μm; **k**: 4 μm; **l**: 100 nm.

A biopsy taken at 15 days of age from the left quadriceps did not show significant diagnostic abnormalities on routine histochemical stains including oxidative stains [Nicotinamide adenine dinucleotide tetrazolium reductase (NADH‐TR), COX and succinate dehydrogenase (SDH)] or fibre typing stains (Figure 1 **a**–**d**). Furthermore, ultrastructural examination only revealed prominent nonmembrane‐bound sarcoplasmic glycogen but no abnormalities of mitochondrial ultrastructure (Figure 1 **e**).

In contrast, a right quadriceps biopsy taken at 12 years showed a number of abnormalities including some small, predominantly angular, fibres (down to approximately 5 μm) (Figure 1 **f**). There were occasional internal nuclei, but these were not a prominent feature. There was no fibre necrosis, regeneration or splitting. There was a fine vacuolation associated with Gömöri‐positive material but no ragged red fibres (Figure 1 **g**). In a few areas, larger deposits of Gömöri‐positive material were noted. There was prominent lipid staining on Oil red O (Figure 1 **h**). There was some coarse staining on the NADH‐TR stain, but staining for SDH and COX was normal (Figure 1 **i**). Fibre typing with immunohistochemistry for fast and slow myosin showed fast fibre predominance. There were small fibres of both types. A few fibres stained for neonatal myosin. We considered the possibility that the abnormality of fibre typing may have represented either a neurogenic component or a selective loss of slow fibres. However, apart from angular atrophic fibres, specific features to distinguish these possibilities were not seen. Histochemical staining for adenylate deaminase showed scattered dense aggregates of staining (Figure 1 **j**). Staining for SERCA1 and SERCA2 showed a fibre‐type pattern but did not reveal significant aggregates. Electron microscopy showed frequent subsarcolemmal TAs composed of parallel collections of concentric double‐walled tubules (Figure 1 **k**,**l**). The aggregates were very frequent but each individually was relatively small, which may explain the difficulty of recognizing them on histochemical stains. The excess lipid noted histochemically was confirmed on electron microscopy, but there were no abnormalities of mitochondrial structure.

Genetic studies were performed with ethical approval from the National Research Ethics Committee London Bloomsbury, UK. In view of the low complex II + III activities in the first biopsy, the *MT‐CYB* and *BCS1L* genes were sequenced; no pathogenic mutations were identified. Full mitochondrial genome sequencing was performed in muscle from the second biopsy and was also normal. A whole exome sequencing approach was then utilised to further investigate presumed autosomal recessive Leigh syndrome, using the Illumina HiSeq platform. Raw fastq files were aligned to the hg19 reference genome using Novoalign version 2.08.03 (Novocraft Technologies Sdn Bhd, Malaysia). For this case sample, as well as a collection of 2600 in‐house control samples processed together, we created gVCF files using the Haplotype Caller module of the Genome Analysis Tool Kit (gatk) version 3.1.1 (Broad Institute, USA). These individual gVCF files were combined into combined gVCF of 100 samples, which were then used for variant calling (using the GenotypeGVCF module of gatk 3.1.1). Variants quality scores were recalibrated according to GATK best practices separately for indels and single nucleotide polymorphisms. Rare variants (defined as an allele frequency <0.5% in‐house control samples) which were nonsynonymous, presumed loss of function or splicing were prioritized. Of 21 388 exonic calls, eight were rare homozygous, and 23 genes contained compound heterozygous calls. In the mitochondrial gene *SERAC1* (serine active site containing 1), encoding a putative phosphatidylglycerol remodelling protein, we identified a known homozygous pathogenic splice mutation c.1403 + 1G>C in exon 14. This mutation leads to a skipping of exon 13 and nonsense mediated mRNA decay [5]. Both parents were heterozygous for this mutation, confirming segregation with disease in this family.

*SERAC1* encodes a phospholipase which has been postulated to be involved in cholesterol trafficking and lipid remodelling at the Mitochondria‐associated endoplasmic reticulum membranes (ER‐MAM) interface [5]. The presence of TAs in our patient's muscle at 12 years but not in his neonatal biopsy suggests that sarcotubular structure has altered in the intervening period, which we hypothesise directly results from inefficient lipid remodelling as a consequence of the *SERAC1* mutations, leading to aggregation of ER constituents. This hypothesis is supported by the presence of TA‐like structures in Chinese hamster ovary cells overexpressing 3‐hydroxy‐3‐methylglutaryl‐coenzymeA reductase, an ER enzyme involved in cholesterol biosynthesis [6]. Delayed formation of TAs in our patient fits with previous observations that TA formation is slow *in vivo*, typically months in mice [7], which may equate to years in humans. Previous reports of EM in patients with *SERAC1* mutations noted abnormal mitochondrial architecture in liver [8] and muscle [5] but not TAs. However, all previously reported muscle biopsies appear to have been performed in infancy or very early childhood [5, 8, 9, 10], which likely explains the absence of TAs in previous cases. Therefore, this is the first report of the natural course of muscle pathology in SERAC1 deficiency. SERAC1 deficiency is one of a growing family of phospholipid biosynthesis and remodelling disorders, which may affect the heart, skeletal muscle, brain or peripheral nerves [11].

The variable RC deficiencies observed in SERAC1 deficiency, with normal enzyme activities in some individuals including the second biopsy in the patient reported here, mean that abnormal RC activities cannot be used as a screening test for this disorder. Rather, the presence of a Leigh‐like encephalopathy with SNHL and 3‐methylglutaconic aciduria should trigger clinical suspicion of 3‐methylglutaconic aciduria with deafness, encephalopathy and Leigh‐like syndrome (MEGDEL syndrome), and sequence analysis of *SERAC1* should be performed. Moreover, our findings suggest that energy deficiency is not the primary pathogenic mechanism in SERAC1 deficiency and that abnormal subcellular aggregate formation as a result of disordered lipid membrane remodelling may be more important in the multisystemic pathogenesis of this disorder, including deafness and progressive encephalopathy. Furthermore, based on the evidence presented here, we suggest abnormal lipoprotein aggregation may be a common pathogenic mechanism in disorders of phospholipid biosynthesis and remodelling.
